# Principles and Practice of Surgery

**Published:** 1848

**Authors:** 


ARTICLE V.

*Principles and Practice of Surgery.* By the late GEO. McCLELLAN, M.D., Edited by his son JOHN B. McCLELLAN, M.D. Philadelphia: Grigg, Elliott, & Co., 1848. pp. 432, octavo. (From the Publishers—for sale by Morrison & Talbott, Indianapolis.)

This is a handsome volume, got up in the very best style, and, as was expected, proves to be a very valuable, though a brief book. The "Principles and Practice of Surgery," comprised, index and all, within four hundred and thirty two pages, makes a very striking contrast with the ponderous works of Velpeau and Chelius, the American edition, of the former of these making 3005, and of the latter 2107 pages.

The work, however, is confessedly incomplete; but the Editor proposes, at some future period, as nearly as possible, to carry out the original design of his illustrious father in its completion, by adding to it a second volume. [E.

